# Orbito-Frontal Cortex Hypometabolism in Children With Post-COVID Condition (Long COVID): A Preliminary Experience

**DOI:** 10.1097/INF.0000000000003578

**Published:** 2022-07-13

**Authors:** Fabrizio Cocciolillo, Daniela Di Giuda, Rosa Morello, Cristina De Rose, Piero Valentini, Danilo Buonsenso

**Affiliations:** From the * Dipartimento di Diagnostica per Immagini, Radioterapia Oncologica ed Ematologia, UOC di Medicina Nucleare, Fondazione Policlinico Universitario Agostino Gemelli IRCCS, Rome, Italy; † Istituto di Medicina Nucleare, Università Cattolica del Sacro Cuore, Roma, Italia; ‡ Department of Woman and Child Health and Public Health, Fondazione Policlinico Universitario A. Gemelli IRCCS, Rome, Italy and; § Global Health Research Institute, Istituto di Igiene, Università Cattolica del Sacro Cuore, Roma, Italy

**Keywords:** long covid, children, brain, PET, orbito-frontal cortex

## Abstract

We describe 3 children with new-onset neurocognitive problems after coronavirus disease 2019 (COVID-19), that showed, at the brain [18F]-fluorodeoxyglucose positron emission tomography/computed tomography, hypometabolism in the left orbito-frontal region. The voxel-wise analysis confirmed a cluster of hypometabolic voxels in this region with a peak at −18/46/−4mm (179 voxels, T-Score 8.1). These findings may explain neurocognitive symptoms that some children develop after COVID-19 and require further investigations

There is increasing recognition that SARS-CoV-2 can not only cause severe acute disease [coronavirus disease 2019 (COVID-19)] but a subgroup of patients may develop a chronic condition that impacts daily functioning for months after the initial infection.^1^ This condition, commonly referred to as long Covid or post-Covid condition (PCC), is well characterized in adults, where there is evidence from several international independent cohorts that up to 30%–50% of the patients can be affected. Recent studies are providing evidence that adult PCC can have immunologic dysfunction^1^ with or without evidence of organ involvement. Although PCC is a multisystem disease, cardiac,^1^ lung,^1^ and more recently brain pathologic findings^2^ at third level imaging studies have been demonstrated.

The latest studies have shown that also a subgroup of children do not fully recover from SARS-CoV-2 infection but develop persisting clinical symptoms, such as fatigue, post-exertional malaise, arthralgia, headache, chest pain and neuropsychiatric problems, including sleep disorders, alterations in mood, memory and concentration.^3^ However, available studies have mostly focused on surveys and self-reported symptoms, whereas investigations on pathophysiology are scarce. Indeed, central nervous system functional imaging may be particularly relevant, since children may develop neurocognitive symptoms and it is still unclear if they have a psychological or a more organic substrate.^4^ Most importantly, recent adult studies have documented functional and morphological changes in the brain of adults affected by COVID-19.^2^

In this study, we present preliminary findings of a small group of children with new, chronic neurocognitive problems never referred before COVID-19, evaluated by the brain [18F]-fluorodeoxyglucose positron emission tomography/computed tomography (^18^F-FDG PET/CT).

## METHODS

### Study Population

This study is part of a larger observational study of children younger than 18 years of age assessed in the out-patient pediatric post-Covid center of our institution, after a microbiologically confirmed diagnosis (based on SARS-CoV-2 detected on nasopharyngeal swab by real time polymerase chain reaction) of SARS-CoV-2 infection. Therefore, we assess both children that fully recovered and those with persistent symptoms after the infection. Specifically, the following definition of long Covid (or PCC) is used in our center, according to a Delphi consensus^5^: “Post-COVID-19 condition occurs in young people with a history of confirmed SARS CoV-2 infection, with 1 or more persisting symptoms for a minimum duration of 12 weeks after initial testing that cannot be explained by an alternative diagnosis. The symptoms have an impact on everyday functioning, may continue or develop after COVID-19 infection, and may fluctuate or relapse over time”.

As part of our protocol (available elsewhere,^6^ we propose brain ^18^F-FDG PET/CT imaging for children with neurocognitive signs or symptoms lasting for more than 3 months and which have a negative impact on their routine. The protocol is approved by the ethics committee of our Institution (ID 3078). Written informed consent was obtained from all participants or legal guardians.

### Brain Imaging

Brain ^18^F-FDG PET/CT scans were carried out using the Biograph mCT64 PET/CT scanner (Siemens Healthineers) in a three-dimensional acquisition mode. Each patient fasted 6 hours before the radiotracer injection. FDG PET acquisition started 40 minutes after a slow intravenous bolus injection of ^18^F-FDG (3.7 MBq/kg), while the subject rested quietly in a dimly-lit and silent room. PET images were reconstructed using an iterative time of flight algorithm with CT-based attenuation correction as well as scatter and random corrections. All scans were acquired and reconstructed with the same technical parameters. After a visual analysis of patient images, a voxel-wise comparison using Statistical Parametric Mapping version 8 (SPM8, Wellcome Department of Cognitive Neurology, London, UK) was performed to identify regional ^18^F-FDG-PET hypometabolism. SPM8 was also employed for the spatial normalization of the Montreal Neurological Institute (MNI) space and spatial smoothing with an 8 mm Gaussian kernel. Patient images were compared with those of 19 healthy controls, selected from a previously gathered database. Statistical analysis was carried out through an unpaired 2-sample *t*-test with the Statistical Parametric Mapping (SPM) contrast set at“−1.1” to detect regional hypometabolism with respect to the control group. The resultant *t* statistic data were created with a threshold of *P* < 0.001 and a cluster extension of 120 voxels.

## RESULTS

Three pediatric patients underwent PET imaging, 1 presenting with chronic olfactory dysfunction and 2 with relevant short-term memory problems, difficulty in concentrating and headaches. The clinical and demographic data are summarized in Table 1.

**TABLE 1.** Main Clinical and Demographic Characteristics of the Three Evaluated Children

	Patient 1	Patient 2	Patient 3
Age	13	14	13
Gender	Female	Male	Female
COVID-19 vaccination before infection	Not vaccinated	Not vaccinated	Not vaccinated
Severity of acute disease	Mild	Mild	Mild
Signs and symptoms			
Fever	Yes	Yes	Yes
Days of fever	7	2	2
Cough	No	No	No
Gastrointestinal	No	No	No
Headache	Yes	No	No
Anosmia	No	No	Yes
Dysgeusia	No	Yes	Yes
Memory problems	No	No	No
Concentration problems	No	No	No
Fatigue	Yes	No	No
Pain (muscle/joints)	Yes	No	No
Rash	Yes	No	No
Distance from acute infection (in days) at PET scan	100	150	185
Time from SARS-CoV-2 infection to onset of neurologic symptoms (weeks)	0	4	6
Persisting symptoms			
Memory Problems	Yes	No	No
Concentration problems	Yes	No	No
Headache	Yes	No	No
Olfactory disfunction	No	Yes*	Yes†
Fatigue	Yes	No	No
Pain (muscle/joints)	Yes	No	No
Rash	Yes	No	No
Other	Post Exertional Malaise	Dysgeusia	Dysgeusia†

* Distortion of smell perception; feeling disgusting his sweat and smells of his best friends; feeling nauseated feeling sweat smell in the gym where he was used to attend since years; nauseated by the smell of his mother’s breath. All these issues limited his confidence in social relationships.

† distortion of most smells. Complete loss of taste of several usual meals including chocolate, with negative impact on daily eating habits and indirect impact on family dynamics.

**FIGURE 1.**
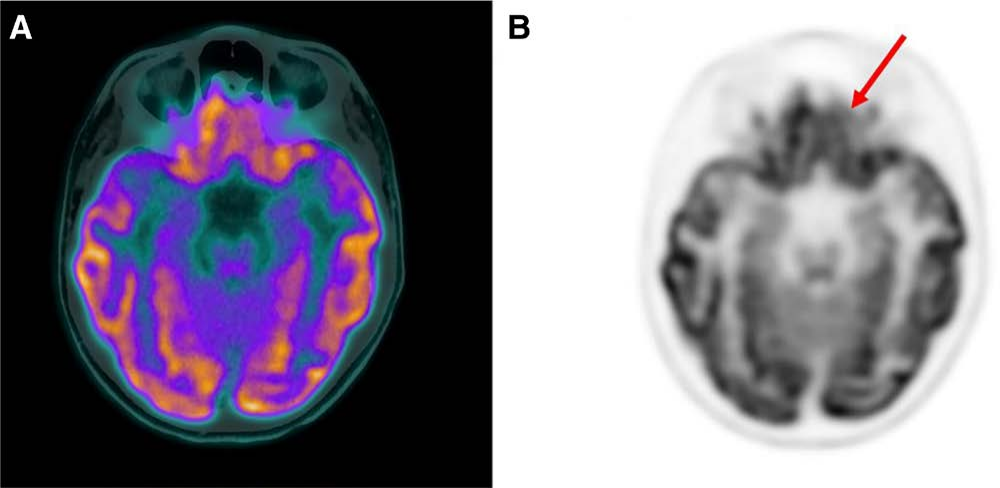
Fused PET/CT (**A**) and PET (**B**) axial slices showing a mild hypometabolism in the left orbitofrontal cortex (red arrow) in patient 1.

**FIGURE 2.**
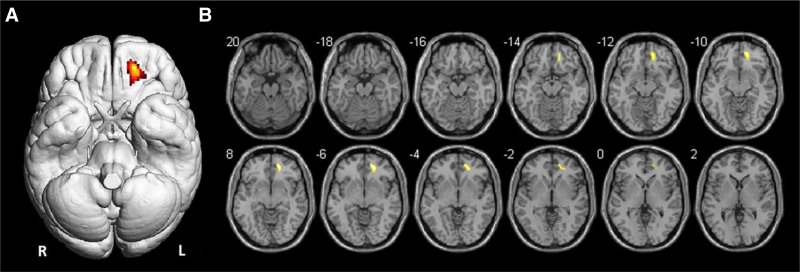
Surface render (A) and axial Magnetic Resonance (MR) slices (B) showing SPM analysis results. The colored areas indicate the locations where the voxel values of the patients are significantly hypometabolic compared to the healthy control group (*P* < 0.001). The *t*-value is represented by the brightness of the color.

The voxel-wise analysis, using *P* values of <0.001, showed lower ^18^F-FDG-PET uptake in the left frontal cortex compared to healthy controls (Figs. 1 and 2). In particular, a cluster of hypometabolic voxels was found in the left orbito-frontal region, with a peak at −18/46/−4 mm (179 voxels, T-Score 8.1).

## DISCUSSION

In this study, we provided preliminary evidence of orbitofrontal cortex hypometabolism in a small group of children experiencing chronic neurological symptoms lasting for more than 3 months after SARS-CoV-2 infection. The metabolic pattern observed in our patients significantly differed from a cohort of the healthy, age-matched control group, as demonstrated by SPM analysis. These data are in line with more robust evidence from neuroradiological follow-up studies of adults assessed after COVID-19. Recent studies, in fact, have documented similar^7^ or more diffuse hypometabolic patterns in adults^8^ and children,^9^ or even more significant changes in brain structure as demonstrated by a UK biobank study.^2^

Our findings are relevant for a number of reasons. So far, there has been debate about the real incidence of pediatric long Covid since studies have documented wide variability in the rate of children presenting with persisting symptoms and some authors have argued that some may suffer from psychological consequences of pandemic restrictions rather than a consequence of the viral infection.^5^ These uncertainties may limit access to diagnostics and care, and also lead to less funding for studying PCC in children. Therefore, objective data of organ involvement, as also previously described in children with abnormal lung perfusions,^6^ provides the scientific community with a new perspective about the real impact of PCC in children. Indeed, this may raise interest towards this condition, which in turn can stimulate funding, research, understanding and, ultimately, pediatric care. Importantly, further studies are needed to better understand which children deserve third-level imaging such as brain ^18^F-FDG PET, also for radiation issues, since a recent review has demonstrated both pathological and normal imaging studies in patients with chronic neuropsychiatric problems.^10^ Lastly, our findings highlight the possibility of a neurotropic effect of the viral infection, either direct or indirect through neuronal transcriptome changes occurring after infection of the neuronal cells. In this regard, Sars-Cov2 infection has been shown to induce the rearrangement of neuronal nuclear architecture.^11^ Such an effect is particularly important in light of the well-established function of the orbitofrontal cortex, which goes beyond the well-established role in taste and smell recognition.^12^ This area of the brain is also implicated in learning and reversing associations of visual and other stimuli, in controlling and correcting reward-related and punishment-related behavior, and thus in emotion.^12^ How this impact the behavior of children and teenagers is still unknown and therefore our findings highlight the need of proper investigation and support of these children, rather than minimizing their complaints. The limited number of children enrolled is an intrinsic limitation of this study, as well as the lack of long-term follow-up. In fact, the long-term implications of these neuroradiologic findings are still unknown, and observational studies will be required to understand it.

In conclusion, we provided evidence of orbitofrontal cortex hypometabolism in children with persistent neuropsychiatric symptoms after SARS-CoV-2 infection, highlighting the need to implement research and raising funds for children with long COVID.
